# Is it time to move to systematic antithrombotic prophylaxis or therapy for all patients with COVID-19 disease?

**DOI:** 10.11604/pamj.2021.39.127.29437

**Published:** 2021-06-15

**Authors:** Sylvain Raoul Simeni Njonnou, Fernando Kemta Lekpa, Eric Balti Vounsia, Jaures Arnaud Noumedem Kenfack, Christian Ngongang Ouankou, Diomede Noukeu Njinkui, Dominique Enyama, Michel Noubom, Simeon Pierre Choukem

**Affiliations:** 1 Department of Internal Medicine and Specialties, Faculty of Medicine and Pharmaceutical Sciences, University of Dschang, Dschang, Cameroon,; 2 The University of Dschang Taskforce for the Elimination of COVID-19 (UNITED#COVID-19), Dschang, Cameroon,; 3 Dschang District Hospital, Dschang, Cameroon,; 4 Douala General Hospital, Douala, Cameroon,; 5 Diabetes Research Center and Department of Internal Medicine, Universiteit Ziekenhuis Brussel, Vrije Universiteit Brussel, Brussels, Belgium,; 6 Endocrine and Diabetes Unit and National Obesity Centre, Department of Internal Medicine, Yaoundé Central Hospital, Yaoundé, Cameroon,; 7 Department of Physiological Sciences and Biochemistry, University of Dschang, Dschang, Cameroon,; 8 Yaoundé University Teaching Hospital, Yaoundé, Cameroon,; 9 Department of Pediatrics, Faculty of Medicine and Pharmaceutical Sciences, University of Dschang, Dschang, Cameroon,; 10 Douala Gynaeco-Obstetric and Paediatric Hospital, Douala, Cameroon,; 11 Department of Microbiology, Haematology and Immunology, Faculty of Medicine and Pharmaceutical Sciences, University of Dschang, Dschang, Cameroon,; 12 Health and Human Development (2HD) Research Network, Douala, Cameroon

**Keywords:** COVID-19, thrombosis, anticoagulation

## To the editors of the Pan African Medical Journal

The coronavirus disease 2019 (COVID-19) has become one of the most tendentious diseases of the 21^st^ century, responsible for more than 2,600,000 deaths worldwide since its declaration in Wuhan (province of Wubei, China) by the end of December 2019. The risk of thrombosis has been described as one of the most prominent features of COVID-19. However, the recommendations for therapeutic anticoagulation are addressed only for severe forms. A recent increase of sudden death in ambulatory patients with COVID-19 in Cameroon prompted us to discuss the possibility to move to anticoagulation for all patients with COVID-9. Thus, we suggest preventive anticoagulation for 14 days for mild forms of COVID-19. For moderate to critical forms, anticoagulation at a curative dose for at least 30 days, and up to 6 months in cases of pulmonary embolism, confirmed or with a high probability. Thus, large-scale randomized clinical trials with indisputable designs are needed.

Dear Sir, the COVID-19 pandemic has become an urgent issue in every country [1]. This disease is multifaceted, ranging from asymptomatic forms in some patients to rapid or sudden death in others. Since its beginning, a relationship between coagulopathy and the SARS-CoV-2 infection has been identified with an increased risk for thromboembolic events [2], particularly among patients with severe forms of the disease [3]. Thus, anticoagulation (prophylactic or therapeutic) has been part of many therapeutic protocols against COVID-19 worldwide. According to these different observations, it is recommended in most protocols that admitted patients should receive prophylactic anticoagulation while patients with severe forms or with a proven venous thromboembolic event (VTE) should receive therapeutic anticoagulation [4]. However, recent literature showed improved survival in patients with moderate or severe disease receiving therapeutic anticoagulation [5, 6]. In Cameroon, anticoagulation is recommended either in prophylaxis for patients with a moderate disease or therapeutically in patients with severe or critical disease (Service Note D31-301/L/MINSANTE/SG dated 08/06/2020).

We recently faced many deaths of young adult patients with COVID-19. Many of these patients died in the second week of their treatment, after taking for five days, with no apparent adverse events, hydroxychloroquine (HCQ) and azithromycin, as recommended by the national protocol for the management of COVID-19 cases in Cameroon. Among them, a 54-year-old male patient with no known comorbidities effectively treated ambulatory with hydroxychloroquine and azithromycin presented a rapidly progressing respiratory distress and died. Another 57-year-old lecturer, without any known comorbidity, presented a fever cough and progressive respiratory distress. Besides being treated with antibiotics, corticosteroids and anticoagulants, he died. In our opinion, these deaths could be certainly attributed to pulmonary embolism. To date, there are no data in the literature supporting anticoagulant prophylaxis or therapeutic anticoagulation in patients with moderate disease. But the new wave of this disease; marked by the occurrence of new variants (English, Brazilian, South African, Indian, Central African and may be other yet to be identified) with an increased reproduction rate and a younger age of admitted patients, is probably associated with increased stimulation of coagulation and more severe forms [7]. Although early prophylactic anticoagulation has been shown to reduce the 30-day in-hospital mortality, therapeutic anticoagulation is associated with a higher risk of bleeding. It is however necessary to draw attention to the fact that these results were obtained by studying the wild type of SARS-CoV-2 [8]. It would be therefore logical, as these new variants gain ground to change our approach to either antithrombotic prophylaxis or therapeutic anticoagulation. Although there is little data in the literature to strongly support these arguments, we were marked by the results of two randomized controlled trials (RCT) on anticoagulation in COVID-19 patients. The first one compared the evolution of patients with positive polymerase chain reaction on a nasopharyngeal sample, treated or not either with antiplatelets and anticoagulants. This study shows reduced mortality in COVID-19 patients taking antiplatelet drugs and low-molecular-weight heparin [9]. In another RCT, COVID-19 patients requiring mechanical ventilation were randomized to receive either therapeutic enoxaparin or the standard anticoagulant thromboprophylaxis. Investigators evaluated the gas exchange over time through the ratio of partial pressure of arterial oxygen (PaO_2_) to the fraction of inspired oxygen (FiO_2_) at baseline, 7, and 14 days after randomization, the time until successful weaning from mechanical ventilation, and the ventilator-free day. In COVID-19 patients receiving therapeutic enoxaparin, there was an improvement of gas exchange and decreases the need for mechanical ventilation in severe COVID-19 [10].

Of course, before prescribing prophylactic and therapeutic anticoagulation to patients with COVID-19, the risk of hemorrhages, the contraindications and the cost should be recorded. Pharmacovigilance of any hemorrhagic event should systematically be assessed with a report sheet included among therapeutic kits for treating COVID-19. But, given the fact that therapeutic anticoagulation has shown to improve the survival rate for patients with severe disease due to wild type of SARS-CoV-2 [6], we suggest expanding its prescription to all patients with COVID-19 or at least to all admitted COVID-19 patient. Although there is no recommendation on the duration of prophylaxis, we suggest, based on our current clinical practice, the use of either low-dose aspirin (80 mg daily) or low-molecular-weight heparin (a daily dose of 40 mg of with standard adjustments for renal insufficiency or obesity can be considered) or a direct oral anticoagulant for a minimum of 14 days for mild forms of COVID-19. For moderate to critical forms, anticoagulation at a curative dose for at least 30 days, and up to 6 months in cases of confirmed pulmonary embolism, or with a high probability (in the setting here confirmation could not be performed). Admittedly, data on anticoagulation in patients with mild forms of COVID-19 are non-existent. But, in view of the observations made in our patients and the proven effectiveness of anticoagulation in patients with moderate to critical forms, we suggest extending its use to mild forms. Such a proposal could, indeed, be evaluated through robust RCT with indisputable design. This will probably represent a challenge for public health policies in sub-Saharan Africa (SSA), in the absence of universal health insurance. A solution for providing systematic therapeutic anticoagulation at least for all hospitalized patients at a lower cost could come from registering these drugs as essential drugs and therefore making them almost free or signing partnerships with the pharmaceutical companies that produce them.

## Conclusion

The recent occurrence of COVID-19 variants is associated with increased reproduction rate, more severe disease, younger age and increased mortality. VTE could be one of the leading causes of these deaths. We suggest preventive antithrombotic therapy in mild forms of COVID-19 and therapeutic anticoagulation for severe to critical ones. The effectiveness of these changes could be evaluated during well conduct large-scale RCT with a clear design.
